# A study to assess the psychosocial aspects of care for cancer patients with COVID-19 at Tata Memorial Hospital, Mumbai, India

**DOI:** 10.3332/ecancer.2021.1330

**Published:** 2021-12-09

**Authors:** Prathepa Jagdish, Manisha Pawar, Anita D’souza, Savitha Goswami, Akshay Patil

**Affiliations:** 1 Department of Nursing, Tata Memorial Hospital, XRXQ+328, Parel East, Parel, Mumbai, Maharashtra 400012, India; 2 Department of Psychology and Counseling, Tata Memorial Hospital, XRXQ+328, Parel East, Parel, Mumbai, Maharashtra 400012, India; 3 Department of Statistics, Tata Memorial Hospital, XRXQ+328, Parel East, Parel, Mumbai, Maharashtra 400012, India

**Keywords:** oncology patients with COVID-19, psycho-social aspects

## Abstract

COVID-19 has more impact on cancer patients due to their immune compromised status. In this study, we tried to understand the impact of cancer patients afflicted with COVID-19 in the physical, emotional, vocational, financial and social domains. The patient caregivers’ problems were also assessed. The investigator tailored the tool and content validity was done by the experts. Total samples were 50 and convenient sampling was used. Descriptive statistics were used and the Shapiro–Wilk’s test was used for normalcy of the variables. The major findings were that the majority belonged to male population with an average annual salary. The diagnosis was hematolymphoid as the main focus compared to breast, bone, gynaecological, gastrointestinal, genitourinary and others. Patients who were receiving chemotherapy were in the majority when compared to radiation, Palliation and surgery. In the physical domain, patients experienced fatigue as a major problem most probably due to the treatment of chemotherapy. The other major problems were loss of smell, breathlessness and loss of appetite. Skin pigmentations were not experienced. In the emotional domain, the major problem was the depression they experienced during COVID-19. In the social domain, financial problems was the most important aspect and access to medication acquirement and transport during the pandemic and job securities were the other problems. Care givers felt social distancing to be a major aspect while looking after patients. They were very uncertain about the prognosis of COVID-19. The Middle age group had more emotional problems.

## Introduction/background

The Tata Memorial Hospital is proud to treat patients with malignancy and also those who have been afflicted with COVID-19. Higher viral loads are associated with a greater risk of death among cancer patients. Cancer patients are susceptible to COVID-19 infection and they are worried about hospitalization which may increase the risk of COVID-19 infections [1]. Among hospitalised COVID-19 patients, those with haematologic malignancies had the highest levels of severe acute respiratory syndrome corona virus [2]. While getting treatment for the physical aspects of cancer, patients should not be neglected about the emotional issues associated with cancer. Psychosocial problems and the needs of cancer patients are increasing due to the pandemic. More and more cancer patients have realised the importance of psychological health, resulting in increased demands for psychological support during the COVID-19 pandemic. Some patients develop chronic anxiety about symptoms and ailments and some experience excessive fears, insomnia, decreased concentration and depression. The psychosocial impacts of COVID-19 on people affected by cancer are multifaceted and likely to have long-lasting consequences. It is important that healthcare providers are equipped to provide patient-centred care during and after this crisis [5]. COVID-19 is expected to be a devastating infection in many patients with solid tumours and hematologic malignancies due to older age, comorbidities and immunocompromised status and therefore should be managed appropriately without jeopardising the curative chance of these patients [6].

Apart from the psychological symptoms, cancer can alter a patient’s life plans, body image, family/social role and financial status [4]. Cancer and the COVID-19 situation has made the patients and their dependents unable to work, resulting in severe financial loss. There is also a lack of access to cancer treatment centers during the pandemic which has caused many cancer patients to worry about their treatment. It is normal to fear these changes but this fear usually lessens over several days or weeks as people adjust to the diagnosis. **One of the best things we as health care professionals can do is to improve their quality of life (QOL) by making them aware about cancer and also COVID-19 so that the information helps them to cope with the situation.** This can make the disease seem less mysterious and frightening. Information from the doctors, nurses and other credible sources can be very helpful in this respect [3].

Considering the increased severity of the disease in these patients, their social, psychological and physical limitations and the number of such patients increasing in society, we strongly believe that it is essential to get a clearer understanding of the unique challenges in protecting patients during this outbreak.

Hence to assess the psycho social aspects, different standardised tools such as Kessler Psychological Distress Scale, Psychosocial Index Scale and Psychosocial assessment tool were assessed. These scales did not address the issues of all domains such as physical, emotional, social, vocational and financial domains. The investigators felt the need to develop a new tool to address all the issues which is a comprehensive tool to understand the Psycho social issues of cancer patients afflicted with COVID-19. Also the investigators have addressed the issues of caregivers of cancer patients with COVID-19 and tried to understand the difficulties faced by the care givers.

Considering the above facts, the nurses undertook the study of the psychosocial aspects of care for cancer patients afflicted with COVID-19 at the Tata Memorial Hospital, Mumbai, India.

## Aims/objectives

### Primary objective

1. The impact of cancer patients afflicted with COVID-19 in the areas of physical and emotional domains.

### Secondary objectives

1. The impact of cancer patients afflicted with COVID-19 in the areas of vocational, financial and social domains.
2. To assess the problems of care givers of cancer patients with COVID-19.
3. To associate the clinical details with the domains of cancer patients afflicted with COVID-19.

### Inclusion criteria

- Adult patients over 18 years with histopathologically proven (malignancy) cancer who developed COVID-19. During the treatment of cancer developed COVID-19. Discharged after COVID-19 treatment from this hospital.
- Oncology patients who had been afflicted with COVID-19. (COVID-19 reports referred from electronic medical records (EMR)).
- Patients who volunteered and complied for the study.

### Exclusion criteria

- Patients unable to follow English and not having telephone accessablility
- Patients with central nervous system involvement and psychiatric conditions.

### Research methodology

Selection of patients using purposive sampling technique.

**Total no of patients – 50**

**Patient selection** – Cancer patients afflicted with COVID-19 from 15 March to 15 November 2020 and registered with the Tata Memorial Hospital.

**Duration of study –** 15 March to 15 November 2020

**Setting –** Cancer patients afflicted with COVID-19 and tested negative and discharged.

**Method of data collection** – An indigenous tool was developed. The tool consisted of Section I – Demographic data. Section II – consisted of domains such as physical, emotional, social, vocational and financial problems. Section III assesses the care givers problem. The tool was developed and validated by seven experts. The criteria used by experts for validation of the tool included item number, relevance, need modification, not relevant, remarks and suggestion. IRB (Institutional Review Board) permission was obtained. The number was 900733.

A telephonic interview was undertaken with the patients and care givers. The duration of the interview was about 20–25 minutes. The questioning was in the English language. After discharge from the hospital, the post period of COVID-19 was around 4–8 weeks. The patients were interviewed after this period.

## Analysis

Demographic data were summarized with descriptive statistics. Continuous data were represented as mean (standard deviation) or median interquartile range and categorical data was reported in counts (percentage), respectively. The Shapiro–Wilk’s test was used to check the normality of each variable. Demographic data were summarised with descriptive statistics.

We used quartiles to divide scores into low, moderate and high levels of QOL of patients. All categorical data were analysed using Chi-square Person’s test.

All analysis was performed by using Statistical Package for the Social Sciences version 25.

## Results

All 50 patients’ study was completed. The criteria of selection of cancer patients were based on histopathological report, COVID-19 reports obtained for EMR both conditions were considered and results were compared. No patients were psychiatric but a psychologist was included in the team for any counseling if required.

Fifty participants consented for the study. The study was comprised 40% females and 60% males. Education ranged from 8% illiterate, 40% of primary education, 32% secondary and 20% of college education. Disease condition ranged from haematological cancers 66.7% and solid tumours 33.3% (haematolymphoid conditions 30%, Bone 12%, breast 6%, head and neck 12%, gastrointestinal 18%, Genitourinary 6%, gynaec 12% and others 4%).

Treatment received by the cancer patients who were admitted at the Tata Memorial Hospital comprised of chemotherapy alone was 46%, adjuvant chemotherapy 2%, palliative chemotherapy 14%, Radiation therapy 6% and surgery was 32%.

### Section I

As shown in Table 1, representing the age group of less than 33 years were 20%, 33–57 years were 64% and more than 57 years were 16%.

Females were 40% and males were 60%.

Hematological cancer patients were 30% and solid cancers were 70%.

Chemotherapy treatment were taken by 34% radiation therapy by 06%, surgery and palliation by 30% each.

### Section II

Table 2 outlined the physical symptoms of the patients experienced. Of all fatigue was experienced by 24%. Loss of smell was experienced by 10% to a large extent. Other symptoms experienced by patients were breathlessness 8% and loss of appetite 6%. The majority 68% did not experience any skin pigmentations at all. Fever was not at all in 2% of the sample.

The second subset consisted of the emotional domain (Table 3). Depression was experienced by 12% of the cancer patients. To a large extent the majority 4% were unable to sleep and 4% could not concentrate on any work.

Table 4 was the social domain. Job insecurity was experienced by 16%. Financial problems was experienced by 28% of the patients. No patients experienced stigma surrounding COVID-19 from their relatives. 20% said transport and medication accessibility was a problem during the time of COVID-19.

### Section III

The next focus was on the care givers (Table 5). 40% experienced social distancing as a major problem while caring for COVID-19 patients with cancer when they were hospitalized. 46% said that they were unable to talk to the patients. 52% said that they were uncertain over many things due to COVID-19 and the related future of their patients.

While the domains were classified as physical, emotional and social domains, it was divided using the Likert scale as patients experienced a low, middle and higher degree of the extent of problems (Table 6). 24% of patients experienced higher life problems in the physical, emotional and social problems and 48% of patients experienced moderate problems in the physical and social domains while only 38% had emotional problems to a moderate extent.

### Section IV

There was no statistical significance with age, sex, diagnosis, and treatment of cancer patients who were afflicted with COVID-19 and the physical domain (Table 7 (a)).

There was no statistical significance with age, sex, diagnosis, and treatment of cancer patients afflicted with COVID-19 and the emotional domain (Table 7 (b)).

## Discussion

The University of Surrey states that the physosocial needs of people affected by cancer are not being adequately met due to the disruption in services caused by COVID-19 [1]. India too needs attention in regards to phychosocial and financial and care givers needs. Many patients experienced depression as a major problem in the emotional domain. The hospital had psychological counsellors on duty to provide psychological counseling services to patients during working hours and psychological clinics remained open during the pandemic. For patients with travel restrictions or fear of being infected by the COVID-19 virus in hospitals, online psychological counselling was provided [4]. We at Tata also carried out online psychological counseling with a psychologist which included guidance on how to relax and meditate, in case of necessity.

Male patients were more afflicted with cancer and COVID-19. Hematolymphoid were the majority compared with the solid tumors. Patients receiving chemotherapy were greater compared to surgery palliation and radiation. Fatigue was the most important physical symptom experienced by the patients with malignancy when compared to loss of smell and breathlessness in the physical domain. Patients said that finance and job were a problem and also had transportation and availability of medication as a greater problem during the times of COVID-19.

Caregivers felt that they were unable to meet their patients which they felt was a major problem during hospitalization. Social distancing and using masks were a difficult task they felt at that time as it was a new procedure. They were also uncertain about many things for life ahead due to COVID-19. There was no statistical significance with the domains and clinical data of cancer patients afflicted with COVID-19.

## Conclusion

Hematolymphoid patients due to chemotherapy reception and their immune suppressed status need to be focused more on COVID-19. Fatigue should be addressed with greater focus. Finance and vocation needs to be handled well by the stakeholders as we have experienced in this pandemic. Nurses not only need to address the issues of the patients but also the care givers.

## Delimitations

A larger population could not be covered due to mitigation.

## Conflicts of interest

No conflicts of interest.

## Funding

No funding was received for this study.

## Figures and Tables

**Table 1.** Demographic details.

Variable	Categories	Frequency	Percentage
Age	<33 years	10	20
	33–57 years	32	64
	>57 years	08	16
Sex	Female	20	40
	Male	30	60
Diagnosis	Hemat	15	30
	Solid	35	70
Treatment	Chemotherapy	17	34
	Radiation therapy	03	06
	Surgery	15	30
	Palliation	15	30

**Table 2.** Physical domain experienced post COVID-19.

Physical symptoms	Not at all present	To a small extent	To some extent	To a moderate extent	To a large extent
Fever	1 (2%)	6 (12%)	21 (42%)	21 (42%)	1 (2%)
Cough	5 (10%)	18 (36%)	15 (30%)	10 (20%)	2 (4%)
Breathlessness	10 (20%)	20 (40%)	13 (26%)	3 (6%)	4 (8%)
Body ache	15 (30%)	26 (52%)	7 (14%)	2 (4%)	0 (0%)
Loss of taste	23 (46%)	19 (38%)	4 (8%)	1 (2%)	3 (6%)
Loss of smell	28 (56%)	12 (24%)	4 (8%)	1 (2%)	5 (10%)
Nausea	23 (46%)	22 (44%)	2 (4%)	2 (4%)	1 (2%)
Vomiting	29 (58%)	16 (32%)	0 (0%)	3 (6%)	2 (4%)
Diarrhea	31 (62%)	17 (34%)	0 (0%)	2 (4%)	0 (0%)
Fatigue	4 (8%)	4 (8%)	14 (28%)	16 (32%)	12 (24%)
Appetite	16 (32%)	14 (28%)	14 (28%)	3 (6%)	3 (6%)
Infection	30 (60%)	10 (20%)	6 (12%)	2 (4%)	2 (4%)
Skin	34 (68%)	13 (26%)	3 (6%)	0 (0%)	0 (0%)

**Table 3.** Emotional domain experienced post COVID-19.

﻿Emotional scale	Not at all present	To a small extent	To some extent	To a moderate extent	To a large extent
Depressed	3 (6%)	14 (28%)	14 (28%)	13 (26%)	6 (12%)
Unable to sleep	7 (14%)	19 (38%)	16 (32%)	6 (12%)	2 (4%)
Irritable	6 (12%)	28 (56%)	10 (20%)	5 (10%)	1 (2%)
Loss of concentration	10 (20%)	23 (46%)	12 (24%)	3 (6%)	2 (4%)

**Table 4.** Social domain experienced post COVID-19.

Social scale	Not at all present	To a small extent	To some extent	To a moderate extent	To a large extent
Job insecurity	3 (6%)	16 (32%)	15 (30%)	8 (16%)	8 (16%)
Finance	6 (12%)	12 (24%)	15 (30%)	3 (6%)	14 (28%)
Stigma to COVID-19	11 (22%)	22 (44%)	14 (28%)	3 (6%)	0 (0%)
Transport	5 (10%)	14 (28%)	15 (30%)	6 (12%)	10 (20%)
Medication	3 (6%)	25 (50%)	9 (18%)	3 (6%)	10 (20%)

**Table 5.** Problems by care givers.

Caregivers scale	Not at all present	To a small extent	To some extent	To a moderate extent	To a large extent
Social distance was a problem	1 (2%)	2 (4%)	9 (18%)	18 (36%)	20 (40%)
Unable to talk	1 (2%)	1 (2%)	12 (24%)	13 (26%)	23 (46%)
Uncertainty over many things.	1 (2%)	2 (4%)	10 (20%)	11 (22%)	26 (52%)

**Table 6.** Domains and degrees of extent of problem.

Domains	Low	Moderate	High
Physical symptoms	14 (28%)	24 (48%)	12 (24%)
Emotional symptoms	19 (38%)	19 (38%)	12 (24%)
Social problems	14 (28%)	24 (48%)	12 (24%)
Caregivers problems	12 (24%)	32 (64%)	6 (12%)

**Table 7 (a).** Association of physical symptoms experienced post COVID-19 with demographic variables.

Variable	Categories	Physical symptoms			*p*-value
		Low	Moderate	High	
Age	<33 years	5 (26.3%)	2 (10.5%)	3 (25%)	0.061
	33–57 years	12 (63.2%)	12 (63.2%)	8 (66.7%)	
	>57 years	2 (10.5%)	5 (26.3%)	1 (8.3%)	
Sex	Female	8 (42.1%)	6 (31.6%)	6 (50%)	0.578
	Male	11 (57.9%)	13 (68.4%)	6 (50%)	
Diagnosis	Hemat	6 (31.6%)	5 (26.3%)	4 (33.3%)	0.345
	Solid	13 (68.4%)	14 (73.7%)	8 (66.7%)	
Treatment	Chemotherapy	4 (21.1%)	11 (57.9%)	2 (16.7%)	0.515
	RT	2 (10.5%)	0 (0%)	1 (8.3%)	
	Surgery	7 (36.8%)	3 (15.8%)	5 (41.7%)	
	Palliation	6 (31.6%)	5 (26.3%)	4 (33.3%)	

**Table 7 (b).** Association of emotional symptoms experienced post COVID-19 with demographic variables.

Variable	Categories	Emotional symptoms			*p*-value
		Low	Moderate	High	
Age	<33 years	5 (35.7%)	2 (8.3%)	3 (25%)	0.484
	33–57 years	5 (35.7%)	20 (83.3%)	7 (58.3%)	
	>57 years	4 (28.6%)	2 (8.3%)	2 (16.7%)	
Sex	Female	6 (42.9%)	9 (37.5%)	5 (41.7%)	0.345
	Male	8 (57.1%)	15 (62.5%)	7 (58.3%)	
Diagnosis	Hemat	6 (42.9%)	5 (20.8%)	4 (33.3%)	0.901
	Solid	8 (57.1%)	19 (79.2%)	8 (66.7%)	
Treatment	Chemotherapy	5 (35.7%)	8 (33.3%)	4 (33.3%)	0.155
	RT	0 (0%)	3 (12.5%)	0 (0%)	
	Surgery	3 (21.4%)	8 (33.3%)	4 (33.3%)	
	Palliation	6 (42.9%)	5 (20.8%)	4 (33.3%)	
